# Feasibility of Using Real-world Data to Emulate Postapproval Confirmatory Clinical Trials of Therapeutic Agents Granted US Food and Drug Administration Accelerated Approval

**DOI:** 10.1001/jamanetworkopen.2021.33667

**Published:** 2021-11-09

**Authors:** Joshua D. Wallach, Audrey D. Zhang, Joshua J. Skydel, Victoria L. Bartlett, Sanket S. Dhruva, Nilay D. Shah, Joseph S. Ross

**Affiliations:** 1 Department of Environmental Health Sciences, Yale School of Public Health, New Haven, Connecticut; 2 Duke University Health System, Durham, North Carolina; 3 Dartmouth-Hitchcock Medical Center, Lebanon, New Hampshire; 4 Yale School of Medicine, New Haven, Connecticut; 5 Section of Cardiology, San Francisco Veterans Affairs Health Care System, San Francisco, California; 6 Department of Medicine, School of Medicine, University of California, San Francisco; 7 Division of Health Care Policy and Research, Mayo Clinic, Rochester, Minnesota; 8 Section of General Internal Medicine, Department of Internal Medicine, Yale School of Medicine, New Haven, Connecticut; 9 National Clinician Scholars Program, Yale School of Medicine, Department of Internal Medicine, New Haven, Connecticut; 10 Department of Health Policy and Management, Yale School of Public Health, New Haven, Connecticut

## Abstract

This cross-sectional study examines the feasibility of using real-world data, such as billing, claims, and electronic health records, to emulate US Food and Drug Administration–required confirmatory clinical trials for the 50 new therapeutic agents that received accelerated approval between 2009 and 2018.

## Introduction

Under the accelerated approval pathway of the US Food and Drug Administration (FDA), therapeutic agents targeting serious or life-threatening diseases can receive approval on the basis of surrogate markers that are reasonably likely to predict clinical benefit conditional on the conduct of postapproval confirmatory trials.^1^ As required by the 21st Century Cures Act of 2016, the FDA has developed guidance on the use of observational methods and real-world data (RWD) (eg, billing, claims, and electronic health record [EHR] data) to generate clinical evidence to fulfill postapproval study requirements.^2^ Prior evaluations have suggested important limitations to feasibly emulating trials using RWD because of difficulties in reliably ascertaining interventions, indications, trial inclusion and exclusion criteria, and primary end points from claims and/or structured EHR data.^3^

A better understanding of the feasibility of emulating FDA-required postapproval trials conducted to verify clinical benefit is critical because these studies often face recruitment challenges, continue to focus on surrogate markers as end points,^1^ and are delayed for years after approval.^1,4^ Therefore, we conducted this cross-sectional study to examine the feasibility of using RWD to emulate FDA-required postapproval confirmatory trials for all new therapeutic agents that received accelerated approval between 2009 and 2018.

## Methods

This study followed the Strengthening the Reporting of Observational studies in Epidemiology (STROBE) reporting guideline. This study did not require institutional review board approval because it was based on publicly available information, in accordance with 45 CFR §46. Informed consent was not needed because no patient data were used.

We used the Drugs@FDA database to identify all FDA-required postapproval confirmatory trials for new molecular entity drugs and biologics that were regulated by the FDA Center for Drug Evaluation and Research and granted accelerated approval between 2009 and 2018.^5^ For all postapproval confirmatory trials designed to verify efficacy, we reviewed study descriptions, ClinicalTrials.gov information, and abstracts of publications to determine the proportion of trials for which the (1) clinical indication, (2) at least 80% of the clinical inclusion and exclusion criteria, (3) the comparator, and (4) the primary end point(s) could be routinely ascertained from RWD using previously described methods (eTable in the Supplement).^3^ These characteristics were considered unlikely to be routinely ascertained from observational data if researchers would find it difficult to develop a computable phenotype using available RWD sources.^3^ The data were analyzed in Microsoft Excel.

## Results

Between 2009 and 2018, the FDA approved 41 new therapeutic agents via the accelerated approval pathway, requiring a total of 50 postapproval confirmatory trials (Table). Twenty postapproval confirmatory trials (40%) were ongoing at the time of accelerated approval.

**Table.** Characteristics of New Therapeutic Agents Granted Accelerated Approval and FDA-Required Postapproval Confirmatory Trials, 2009-2018

Characteristic	No. (%)
**Therapeutic agent characteristics**	41 (100)
Class	
Drug	30 (73)
Biologic	11 (27)
Therapeutic area	
Cancer and hematology	33 (80)
Other	8 (20)
Priority review	
Yes	37 (90)
No	4 (10)
Fast track	
Yes	24 (59)
No	17 (41)
Breakthrough therapy	
Yes	21 (51)
No	12 (29)
NA^a^	8 (20)
Orphan drug designation	
Yes	38 (93)
No	3 (7)
**FDA-required postapproval confirmatory trial characteristics**	50 (100)
Type of postapproval confirmatory trial	
New clinical trial	27 (54)
Complete or submit results	20 (40)
Other^b^	3 (6)
Trial focus	
Efficacy	32 (64)
Efficacy and safety	18 (36)
Randomization	
Yes	43 (86)
No	1 (2)
NA (single group assignment)	6 (12)
Allocation concealment	
Double or higher	18 (36)
Single	0
None	29 (58)
Unclear^c^	3 (6)
No. of study patients, median (IQR)	424 (295-545)

Abbreviations: FDA, US Food and Drug Administration; NA, not applicable.

^a^ Therapeutic agents approved before the origination of the breakthrough therapy designation on July 9, 2012.

^b^ Includes postmarketing requirements for longer-duration trial follow-up, revisions of ongoing trials, and submission of results from new and completed trials.

^c^ All 3 trials with unclear allocation concealment were randomized trials.

Among the 50 postapproval confirmatory trials, 12 (24%) had a clinical indication that could be routinely ascertained from claims and/or structured EHR data, whereas 38 (76%) required nonroutinely ascertainable disease severity or treatment-related qualifiers (Figure). Of the 46 trials for which clinical inclusion and exclusion criteria were available on ClinicalTrials.gov, 2 (4%) had at least 80% of their criteria that could be routinely ascertained. Of the 41 trials with a comparator arm, 22 (54%) used active comparators, all of which could be ascertained using RWD. Of the 49 trials for which primary end point information was available, 20 (40%) had at least 1 end point that could be routinely ascertained from RWD. Overall, none of the FDA-required postapproval confirmatory trials had all of the following: (1) a clinical indication, (2) at least 80% of the clinical inclusion and exclusion criteria, (3) a comparator, and (4) at least 1 primary end point that could be routinely ascertained from RWD.

**Figure.**
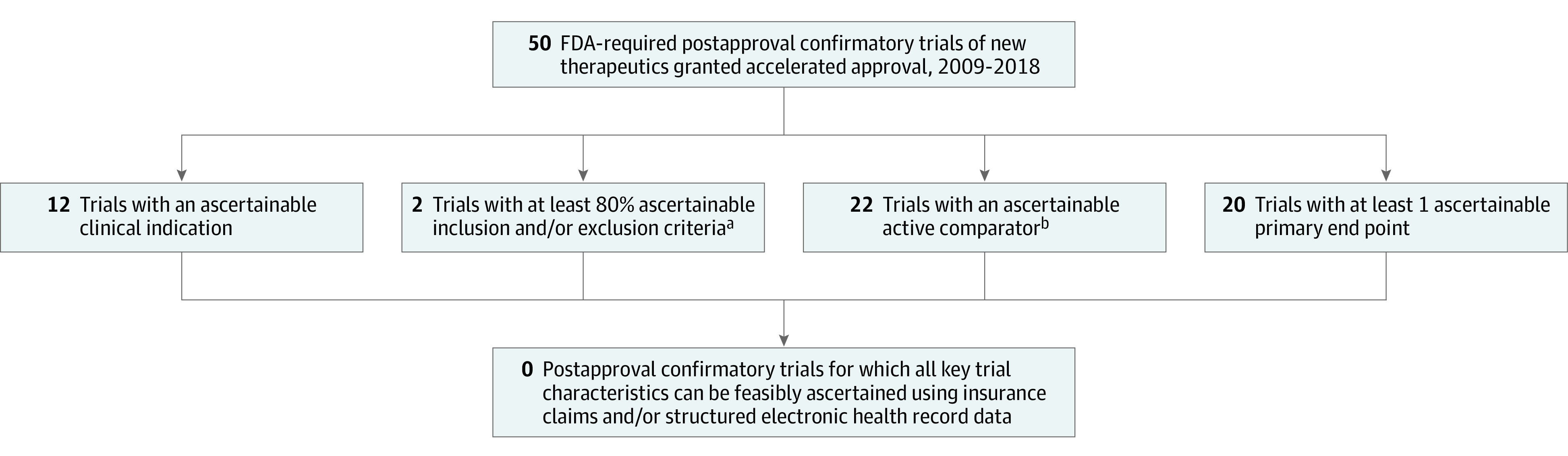
Study Flowchart Flowchart demonstrates the feasibility of using real-world data to emulate 50 US Food and Drug Administration (FDA)–required postapproval confirmatory trials of new therapeutic agents granted accelerated approval between 2009 and 2018. ^a^There were 4 trials without clearly defined inclusion and exclusion criteria (no ClinicalTrials.gov registration). ^b^There were 41 trials with a comparator arm. The “active” designation includes a standard of care comparator, an active comparator, and a standard of care with an active comparator.

## Discussion

The findings of this cross-sectional study suggest that none of the 50 FDA-required postapproval confirmatory trials for therapeutic agents granted accelerated approval between 2009 and 2018 could have been feasibly emulated using currently available claims and/or structured EHR data. In particular, the narrowly defined indications and strict inclusion and exclusion criteria of the FDA-required postapproval confirmatory trials precluded emulation using RWD. Although RWD can be used to ascertain clinical outcomes for real-world populations receiving therapeutic agents, our findings suggest that current observational methods and RWD can complement, but are unlikely to replace, postapproval confirmatory trial requirements.

This study had some limitations. Certain determinations were subjective, we did not consider the relative importance of an individual criterion, and we did not consider accelerated approvals for supplemental indications. Our findings suggest that to use RWD for regulatory evaluations, initiatives are necessary to standardize data elements across different facilities, clinicians, and EHRs.^6^

## Notes

Supplement. **eTable.** Postapproval Confirmatory Trial Emulation Methods
