# Supplementary material for: Feasibility of Using Real-world Data to Emulate Postapproval Confirmatory Clinical Trials of Therapeutic Agents Granted US Food and Drug Administration Accelerated Approval
Source: JAMA Netw Open. 2021 Nov 9;4(11):e2133667. doi: 10.1001/jamanetworkopen.2021.33667 (PMC8579227; doi:10.1001/jamanetworkopen.2021.33667)
Supplement: Supplement. — eTable. Postapproval Confirmatory Trial Emulation Methods [file jamanetwopen-e2133667-s001.pdf]

Supplementary Online Content

Wallach JD, Zhang AD, Skydel JJ, et al. Feasibility of using real-world data to emulate postapproval confirmatory clinical trials of therapeutic agents granted US Food and Drug Administration accelerated approval. *JAMA Netw Open*. 2021;4(11):e2133667. doi: 10.1001/jamanetworkopen.2021.33667

eTable. Postapproval Confirmatory Trial Emulation Methods

This supplementary material has been provided by the authors to give readers additional information about their work.

© 2021 Wallach JD et al. *JAMA Network Open*.

eTable. Postapproval Confirmatory Trial Emulation Methods

**Postmarketing Requirement description**	**Trial Characteristics[a]**		**Can be feasibly ascertained using insurance claims and/or electronic health record data?**
CONFIRMATORY TRIAL - A randomized phase 3 trial of SGN-35 (brentuximab vedotin) in combination with AVD versus ABVD as frontline therapy in patients with advanced Hodgkin Lymphoma. Enrollment of at least 880 patients is expected with a primary end point of progression free survival determined by an independent blinded review facility. Overall survival is a key secondary end point.	Clinical Indication[b]	Hodgkin lymphoma, stage III or IV	No, cannot ascertain cancer stage
	Inclusion/exclusion criteria[c]	Inclusions:<br>1. Treatment-naïve participants with Ann Arbor Stage III or IV HL<br>2. Histologically confirmed classical Hodgkin Lymphoma (HL) according to the current World Health Organization (WHO) classification.<br>3. Eastern Cooperative Oncology Group (ECOG) performance status less than or equal to (<=) 2<br>4. Bidimensional measurable disease as documented by radiographic technique per the International Working Group Revised Criteria for Response Assessment for Malignant Lymphoma<br>Exclusion:<br>5. Nodular lymphocyte predominant Hodgkin lymphoma<br>6. Cerebral/meningeal disease, including signs and symptoms of progressive multifocal leukoencephalopathy (PML)<br>7. Sensory or motor peripheral neuropathy<br>8. Prior immunosuppressive chemotherapy, therapeutic radiation, or any immunotherapy within 12 weeks of first study drug dose<br>9. Known human immunodeficiency virus (HIV) positive<br>10. Known hepatitis B surface antigen-positive, or known or suspected active hepatitis C infection.	No, at least 3 criteria cannot be ascertained (#'s 1, 3, 4)
	Clinical Indication	Hodgkin lymphoma, stage III or IV	No, cannot ascertain cancer stage
	End point(s)	Modified Progression-free Survival (mPFS) Per Independent Review Facility (IRF)<br>[Time Frame: Baseline until PD or death or receipt of any subsequent anticancer therapy for HL after completion of frontline therapy (approximately up to 4 years)]	No, cannot ascertain progression-free survival

AVD, doxorubicin [Adriamycin], vinblastine, and dacarbazine; ABVD, doxorubicin [Adrianmycin], bleomycin, vinblastine, and dacarbazine.

[a] Active comparators were considered not likely to be ascertained from observational data if they lacked a National Drug Code.

[b] Indications were considered not likely to be routinely ascertained if researchers would find it difficult to develop a computable phenotype using structured data, including diagnostic billing codes, procedure codes, vital signs, laboratory test results, and clinical imaging. We did not evaluate the ability to emulate trials if manual or natural language processing methods were applied to unstructured clinical notes.

[c] We did not consider non-clinical inclusion/exclusion criteria (e.g., evidence of informed consent).

© 2021 Wallach JD et al. *JAMA Network Open*.
